# Prediction of myopia development among Chinese school-aged children using refraction data from electronic medical records: A retrospective, multicentre machine learning study

**DOI:** 10.1371/journal.pmed.1002674

**Published:** 2018-11-06

**Authors:** Haotian Lin, Erping Long, Xiaohu Ding, Hongxing Diao, Zicong Chen, Runzhong Liu, Jialing Huang, Jingheng Cai, Shuangjuan Xu, Xiayin Zhang, Dongni Wang, Kexin Chen, Tongyong Yu, Dongxuan Wu, Xutu Zhao, Zhenzhen Liu, Xiaohang Wu, Yuzhen Jiang, Xiao Yang, Dongmei Cui, Wenyan Liu, Yingfeng Zheng, Lixia Luo, Haibo Wang, Chi-Chao Chan, Ian G. Morgan, Mingguang He, Yizhi Liu

**Affiliations:** 1 State Key Laboratory of Ophthalmology, Clinical Research Center for Ocular Disease, Zhongshan Ophthalmic Centre, Sun Yat-sen University, Guangzhou, China; 2 School of Public Health, Sun Yat-sen University, Guangzhou, China; 3 School of Mathematics, Sun Yat-sen University, Guangzhou, China; 4 Zhongshan School of Medicine, Sun Yat-sen University, Guangzhou, China; 5 UCL Institute of Ophthalmology, University College London and Moorfields Eye Hospital, London, United Kingdom; 6 First Affiliated Hospital of Sun Yat-sen University, Guangzhou, China; 7 Laboratory of Immunology, National Eye Institute, National Institutes of Health, Bethesda, Maryland, United States of America; 8 ARC Centre of Excellence in Vision Science, Research School of Biology, College of Medicine, Biology and Environment, Australian National University, Canberra, Australian Capital Territory, Australia; 9 Centre for Eye Research Australia, University of Melbourne, Royal Victorian Eye and Ear Hospital, East Melbourne, Victoria, Australia; University of California San Francisco, UNITED STATES

## Abstract

**Background:**

Electronic medical records provide large-scale real-world clinical data for use in developing clinical decision systems. However, sophisticated methodology and analytical skills are required to handle the large-scale datasets necessary for the optimisation of prediction accuracy. Myopia is a common cause of vision loss. Current approaches to control myopia progression are effective but have significant side effects. Therefore, identifying those at greatest risk who should undergo targeted therapy is of great clinical importance. The objective of this study was to apply big data and machine learning technology to develop an algorithm that can predict the onset of high myopia, at specific future time points, among Chinese school-aged children.

**Methods and findings:**

Real-world clinical refraction data were derived from electronic medical record systems in 8 ophthalmic centres from January 1, 2005, to December 30, 2015. The variables of age, spherical equivalent (SE), and annual progression rate were used to develop an algorithm to predict SE and onset of high myopia (SE ≤ −6.0 dioptres) up to 10 years in the future. Random forest machine learning was used for algorithm training and validation. Electronic medical records from the Zhongshan Ophthalmic Centre (a major tertiary ophthalmic centre in China) were used as the training set. Ten-fold cross-validation and out-of-bag (OOB) methods were applied for internal validation. The remaining 7 independent datasets were used for external validation. Two population-based datasets, which had no participant overlap with the ophthalmic-centre-based datasets, were used for multi-resource validation testing. The main outcomes and measures were the area under the curve (AUC) values for predicting the onset of high myopia over 10 years and the presence of high myopia at 18 years of age. In total, 687,063 multiple visit records (≥3 records) of 129,242 individuals in the ophthalmic-centre-based electronic medical record databases and 17,113 follow-up records of 3,215 participants in population-based cohorts were included in the analysis. Our algorithm accurately predicted the presence of high myopia in internal validation (the AUC ranged from 0.903 to 0.986 for 3 years, 0.875 to 0.901 for 5 years, and 0.852 to 0.888 for 8 years), external validation (the AUC ranged from 0.874 to 0.976 for 3 years, 0.847 to 0.921 for 5 years, and 0.802 to 0.886 for 8 years), and multi-resource testing (the AUC ranged from 0.752 to 0.869 for 4 years). With respect to the prediction of high myopia development by 18 years of age, as a surrogate of high myopia in adulthood, the algorithm provided clinically acceptable accuracy over 3 years (the AUC ranged from 0.940 to 0.985), 5 years (the AUC ranged from 0.856 to 0.901), and even 8 years (the AUC ranged from 0.801 to 0.837). Meanwhile, our algorithm achieved clinically acceptable prediction of the actual refraction values at future time points, which is supported by the regressive performance and calibration curves. Although the algorithm achieved balanced and robust performance, concerns about the compromised quality of real-world clinical data and over-fitting issues should be cautiously considered.

**Conclusions:**

To our knowledge, this study, for the first time, used large-scale data collected from electronic health records to demonstrate the contribution of big data and machine learning approaches to improved prediction of myopia prognosis in Chinese school-aged children. This work provides evidence for transforming clinical practice, health policy-making, and precise individualised interventions regarding the practical control of school-aged myopia.

## Abstract 2

Therapies to control myopia progression confer significant side effects and should be targeted to those at highest risk. Here, Yizhi Liu and colleagues report a machine learning algorithm that predicts the progression of myopia, into early adulthood, among Chinese school-aged children.

## Author summary

**Why was this study done?:**

- Myopia has reached epidemic levels among young adults in East and Southeast Asia, affecting an estimated 80%–90% of high school graduates, with approximately 20% of them having high myopia. Various interventions, including atropine eyedrops and orthokeratology, have been proposed to control myopia progression; however, these approaches confer significant side effects. Identifying those at greatest risk who should undergo targeted therapy is the most important clinical challenge faced by ophthalmologists and optometrists.
- Electronic medical records provide large-scale real-world clinical data for use in developing clinical decision systems. Taking school-aged myopia, the most prevalent eye disease, as an example, it would be of great value to use ophthalmic-centre-based electronic medical records to develop a big-data-driven clinical prediction algorithm based on machine learning algorithms.

**What did the researchers do and find?:**

- This study analysed 687,063 longitudinal electronic medical records from the largest ophthalmic centres in China and developed and validated individualised prediction models for myopia prediction based on machine learning techniques.
- Our model predicted spherical equivalent and onset of high myopia at 18 years of age at a clinically acceptable accuracy and as early as 8 years in advance.

**What do these findings mean?:**

- The algorithm, which was trained and validated using a large real-world dataset, was able to predict the presence of high myopia with clinically acceptable accuracy among Chinese school-aged populations.
- Large-scale, long-term electronic medical records and machine learning algorithms provide unique opportunities for the development of prediction models for progressive diseases, such as myopia in school-aged children.
- Our findings have great potential to change current approaches used to manage school myopia by paediatric and general ophthalmologists as well as general practitioners and optometrists, who are often the first point of care.

## Introduction

Myopia, the most common visual impairment in children, has increased markedly in Chinese school-aged children in recent years. This “myopia boom” is a significant international public concern, impacting study performance and daily life [1]. The risk of children developing high myopia has become a great concern among parents [2], with thousands of students seeking care at optometric and ophthalmic clinics annually in China. This creates an enormous burden for the healthcare system but provides an unprecedented opportunity to collect large-scale real-world clinical data that are unified and reliable.

Big data available from service providers contain valuable “signals” for authentic disease progression and prognosis; however, the analysis of these data is challenging because such data are often contaminated by various types of “noise”, given that the data are not collected in a controlled research setting [3]. Machine learning offers a ubiquitous and indispensable method to solve these complexities of data noise and heterogeneity, having the capacity to combine enormous numbers of predictors in a non-linear and highly interactive way [4].

This study is a data-and-algorithm-driven analysis of more than half a million optometry records and data derived from long-term population-based cohort studies in China. The goal was to build a prediction algorithm based on machine learning techniques to uncover the key determinants of high myopia and to predict, as early and as accurately as possible, the development of high myopia in adulthood. The performance of the algorithm was validated using multi-source datasets from independent ophthalmic centres and population-based research cohorts. The results provide evidence for health policy-making regarding the practical control of school-age myopia and precise individual interventions.

## Methods

### Data source

**Fig 1:**
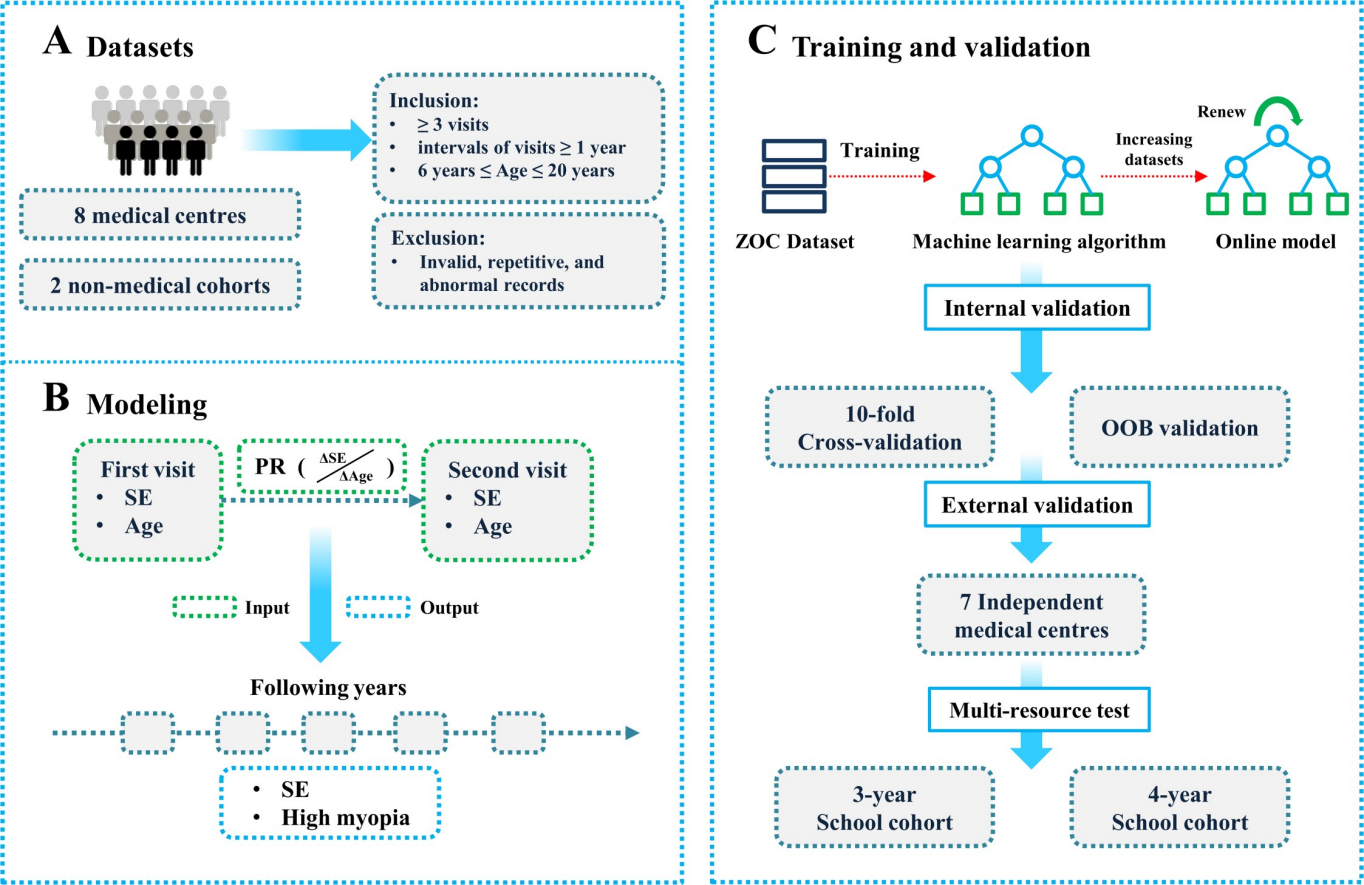
Overall study pipeline. (A) Eight ophthalmic centres and 2 non-medical (population-based) cohorts were included. To focus on the school-aged population, only individuals aged from 6 to 20 years at the initial examination, with ≥3 visits at ≥1-year intervals were included in the analysis. (B) Candidate predictors included age at examination, SE, and annual progression rate. Using these predictors, the algorithm was used to predict SE and whether patients will progress to high myopia in the subsequent 10 years (with each year as a predictive time point). (C) We used the random forest method of machine learning to establish a prediction algorithm. All records from ZOC were used as the training set. Ten-fold cross-validation and OOB methods were applied for internal validation. The remaining records from the other 7 centres and 2 independent population-based datasets were used for external validation and the multi-resource test, respectively. PR, annual progression rate; OOB, out-of-bag; SE, spherical equivalent; ZOC, Zhongshan Ophthalmic Centre.

**Fig 2:**
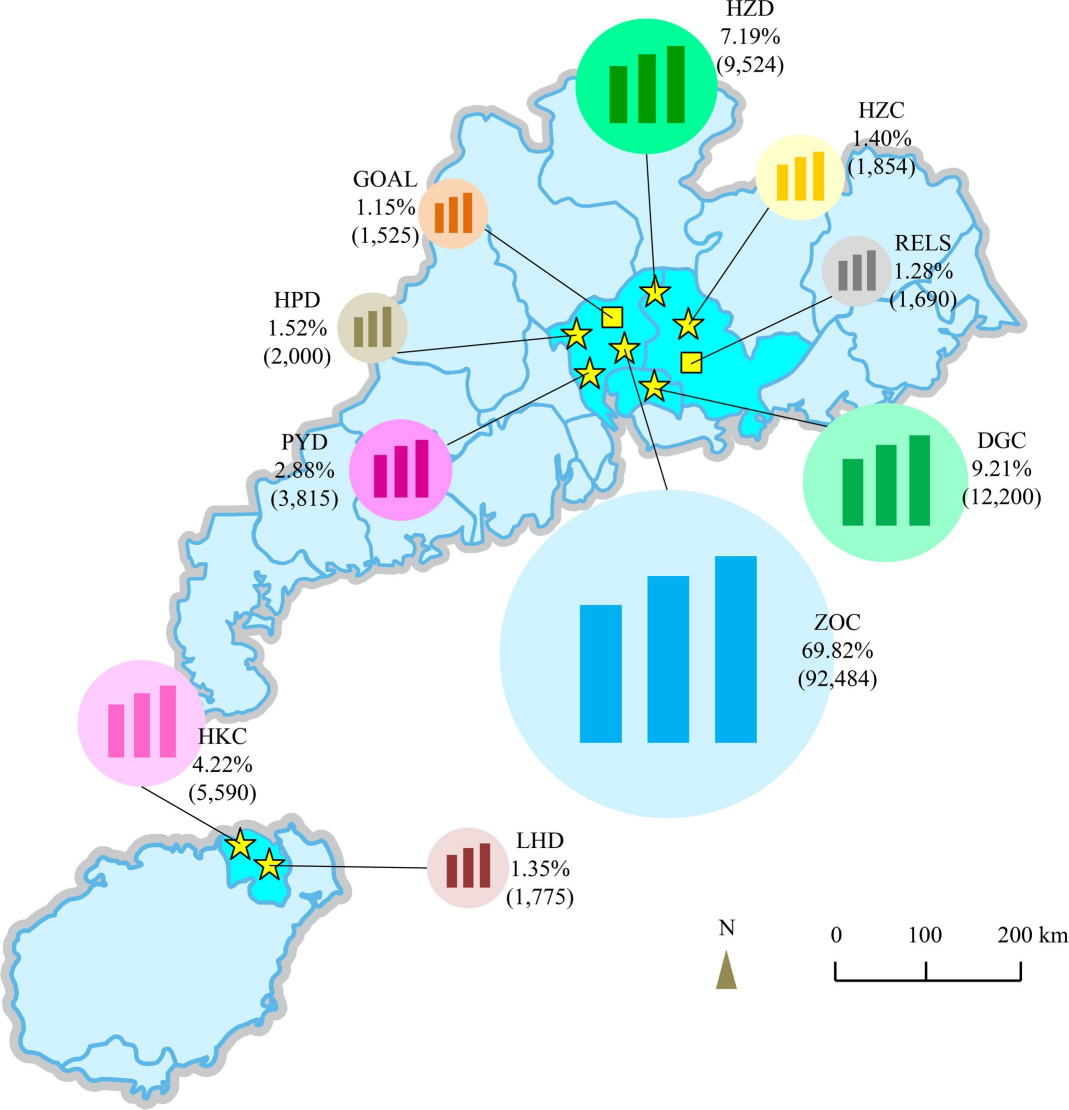
Overall distribution of the study population. A total of 132,457 participants were recruited from 8 ophthalmic centres—4 centres in Guangzhou (Zhongshan Ophthalmic Centre [ZOC, 92,484, 69.82%], the Haizhu Optometry Department [HZD, 9,524, 7.19%], the Huangpu Optometry Department [HPD, 2,000,1.52%], and the Panyu Optometry Department [PYD, 3,815, 2.88%]) and 4 centres outside of Guangzhou (the Dongguan Guangming Ophthalmic Hospital [DGC, 12,200, 9.21%], the Optometry Centre in Huizhou City [HZC, 1,854, 1.40%], the Haikou Longhua Optometry Department [LHD, 1,775, 1.35%], and the Xiuying Optometry Department in Haikou City [HKC, 5,590, 4.22%])—and 2 non-medical (population-based) cohorts (the Guangzhou Outdoor Activity Longitudinal Trial [GOAL, 1,525, 1.15%] and the Refractive Error Longitudinal Study [RELS, 1,690, 1.28%]). The 8 ophthalmic centres and 2 cohorts are in South China and Southeast Asia and provided a representative big data sample.

A summary of study procedures is presented in Fig 1. Eight ophthalmic centres were included in the study, including Zhongshan Ophthalmic Centre (ZOC), the Haizhu Optometry Department (HZD), the Huangpu Optometry Department (HPD), the Panyu Optometry Department (PYD), the Dongguan Guangming Ophthalmic Hospital (DGC), the Optometry Centre in Huizhou City (HZC), the Haikou Longhua Optometry Department (LHD), and the Xiuying Optometry Department in Haikou City (HKC). This study also included 2 datasets collected from population-based cohort studies: the Guangzhou Outdoor Activity Longitudinal Trial (GOAL) [5] and the Refractive Error Longitudinal Study (RELS). These 8 ophthalmic centres and 2 cohorts from South China collectively composed a representative medical big data sample for children of Chinese ethnicity. This sample could be generalisable to Chinese children living in Hong Kong, Taiwan, and Singapore, where myopia is similarly a common public health problem in children. The geographical locations and a detailed description of the study population are presented in Fig 2, S1 Table, and S1 Text.

### Ethics statement

The study adhered to the tenets of the Declaration of Helsinki, and approval for the study protocol was obtained from the Institutional Review Board/Ethics Committee of Sun Yat-sen University (Guangzhou, China). All of the datasets used throughout the study were deidentified prior to transfer to the study investigators.

### Dataset preparation

We extracted data from electronic medical record systems collected between January 1, 2005, and December 30, 2015, at the optometry service of 8 participating ophthalmic centres. To focus on the school-aged population, only individuals aged from 6 to 20 years at the initial examination and with ≥3 visits at ≥1-year intervals were included in the current analysis.

### Predictors and outcomes

Predictors included age at examination, spherical equivalent (SE), and annual progression rate. Cycloplegic refraction was performed according to a standard protocol in each centre. The right eye was arbitrarily chosen to represent a specific individual.

Using these predictors, we aimed to develop an algorithm to predict SE and presence of high myopia in the subsequent 10 years (with each year as a predictive time point). The presence of high myopia was defined as a SE ≤ −6.0 dioptres.

### Algorithm development and validation

Electronic medical records from ZOC were used as the training dataset, and 10-fold cross-validation and out-of-bag (OOB) validation methods [6] were applied for internal validation (details are provided in S2 Text). Meanwhile, a methodological comparison of random forest and other conventional algorithms (generalised estimating equation [7] and mixed-effects model [8]; details are provided in S3 Text) was performed using the average performance of the cross-validation. A complete algorithm was trained on the entire ZOC dataset prior to external validation (variable contributions in S1 Fig).

The refraction data records from the other 7 centres were used for external validation. All individuals from the 7 centres with available refraction records at 18 years of age and with at least 2 visits (≥1-year interval) were included. These records were used to explore the accuracy of prediction at a given time before 18 years of age. Two population-based longitudinal cohorts were used for the multi-resource test.

### Random forest algorithm

Random forest is an ensemble learning method that operates by constructing a multitude of decision trees at training time and outputting the class that is the mode of the classes (classification) or mean prediction (regression) of the individual trees. Here, we employed the random forest algorithm for the development of the prediction algorithm, which was established in the BrainWave machine learning module [9]. The R randomForest package, which implements Breiman’s classic algorithm, was used to fit the random forest model [10].

Each decision tree in the random forest was built using a bootstrap sample with replacement from the original data. This bootstrap aggregation and random feature selection helped reduce the variance of the algorithm and avoided over-fitting. Consequently, in the random forest algorithm, cross-validation is performed internally, which can be just as effective as using a separate test set to estimate the generalisation error of the training set. Moreover, the random forest algorithm can be used to evaluate the variables in a dataset and to provide a graphical display to assess the importance of each variable.

The 2 random forest parameters, mTry (i.e., the number of input variables randomly chosen at each split) and nTree (i.e., the number of trees to grow for each forest), were set to 2 (square root of 5 features) and 500, respectively. In each tree, each feature received a variable importance (VIMP) score, which can be used to rank and select relatively important features.

Regarding the regression analysis, the most widely used VIMP score of a feature is the average percent increase in the OOB mean square error (MSE) as a result of randomly permuting the OOB feature values [11]. The MSE is the mean of the squared regression residuals, and the VIMP score of a feature indicates its overall predictive ability for the regression. Regarding the classification analysis, the error rate is the proportion of misclassified samples of the total number of samples, and the VIMP score of a feature indicates its overall predictive ability for the classification.

### Evaluation metrics

Three evaluation metrics—the coefficient of determination (*R*^2^), the root mean square error (RMSE), and the mean absolute error (MAE)—were used to assess the performance of the regression algorithm in predicting a targeted SE [12]. *R*^2^ can be expressed as

$$R^{2}=1-\mathrm{MSE}/\mathrm{Var}(y)$$

where MSE is the same as noted above and Var(*y*) is the variance of the actual value. The RMSE is the square root of the MSE, which penalises large errors but has the same units as the original response variable being predicted; thus, its magnitude is more easily interpreted. The MAE measures the forecast accuracy by averaging the absolute values of the residuals. The MAE is expressed in the same units as the original response variable and provides an average size of the “miss”, regardless of the direction. This variable can be expressed as

$$\mathrm{MAE}=\frac{1}{n}\sum_{i=1}^{n}\left|y_{i}-{\hat{y}}_{i}\right|$$

where *y*_*i*_ is the actual value, and ${\hat{y}}_{i}$ is the predicted value. These 3 evaluation metrics were calculated for the different predicted target times of each algorithm.

For classification performance, the receiver operating characteristic (ROC) curves and area under the curve (AUC) values were calculated as a comprehensive evaluation. All analyses were performed using R statistical software version 3.2.4 [13].

## Results

### Overall study population

A description of the study population is displayed in Table 1. In total, 687,063 longitudinal electronic medical records of 129,242 individuals from 8 ophthalmic centres and 17,113 follow-up records for 3,215 participants in population-based cohorts were included in the analysis. A total of 517,949 records from ZOC were used as the training set (the follow-up duration ranged from 2 to 11 years, mean ± SD 4.6 ± 1.9 years). The datasets of the remaining 7 centres (169,114 records; the follow-up duration ranged from 2 to 11 years, mean ± SD 5.2 ± 2.1 years) were used for external validation, and the records from the 2 population-based cohorts (17,113 records; the follow-up duration ranged from 3 to 5 years, mean ± SD 4.1 ± 1.2 years) were used for multi-resource validation testing.

**Table 1:** Overall characteristics.

Characteristic	Training set	Validation set	Multi-source set
Number of persons	92,484	36,758	3,215
Number of records	517,949	169,114	17,113
Female, number (%)	49,215 (53.2)	19,247 (52.4)	1,476 (45.9)
Follow-up, mean ± SD (range), years	4.6 ± 1.9 (2–11)	5.2 ± 2.1 (2–11)	4.1 ± 1.2 (3–5)
Age at first visit, mean ± SD, years	8.1 ± 1.5	7.4 ± 1.2	6.9 ± 0.5
Age at last visit, mean ± SD, years	13.8 ± 4.9	14.6 ± 4.1	10.1 ± 1.3
SE at the first visit, mean ± SD, dioptres	−1.5 ± 1.8	−0.8 ± 1.5	0.8 ± 0.9
SE at the last visit, mean ± SD, dioptres	−3.6 ± 1.7	−3.9 ± 1.6	−1.3 ± 2.1

### Internal validation

For the comparative analysis, the random forest algorithm outperformed the generalised estimating equation and the mixed-effects model in the detection of high myopia (S2 Fig). Therefore, all subsequent analyses were conducted based solely on the random forest algorithm. For classification, AUC values more than 0.9 indicated excellent performance, and values from 0.8 to 0.9 indicated good performance; MAE within ±0.75 dioptres was considered clinically acceptable accuracy (i.e., clinically acceptable prediction) based on the measurement variations of refraction [14].

**Fig 3:**
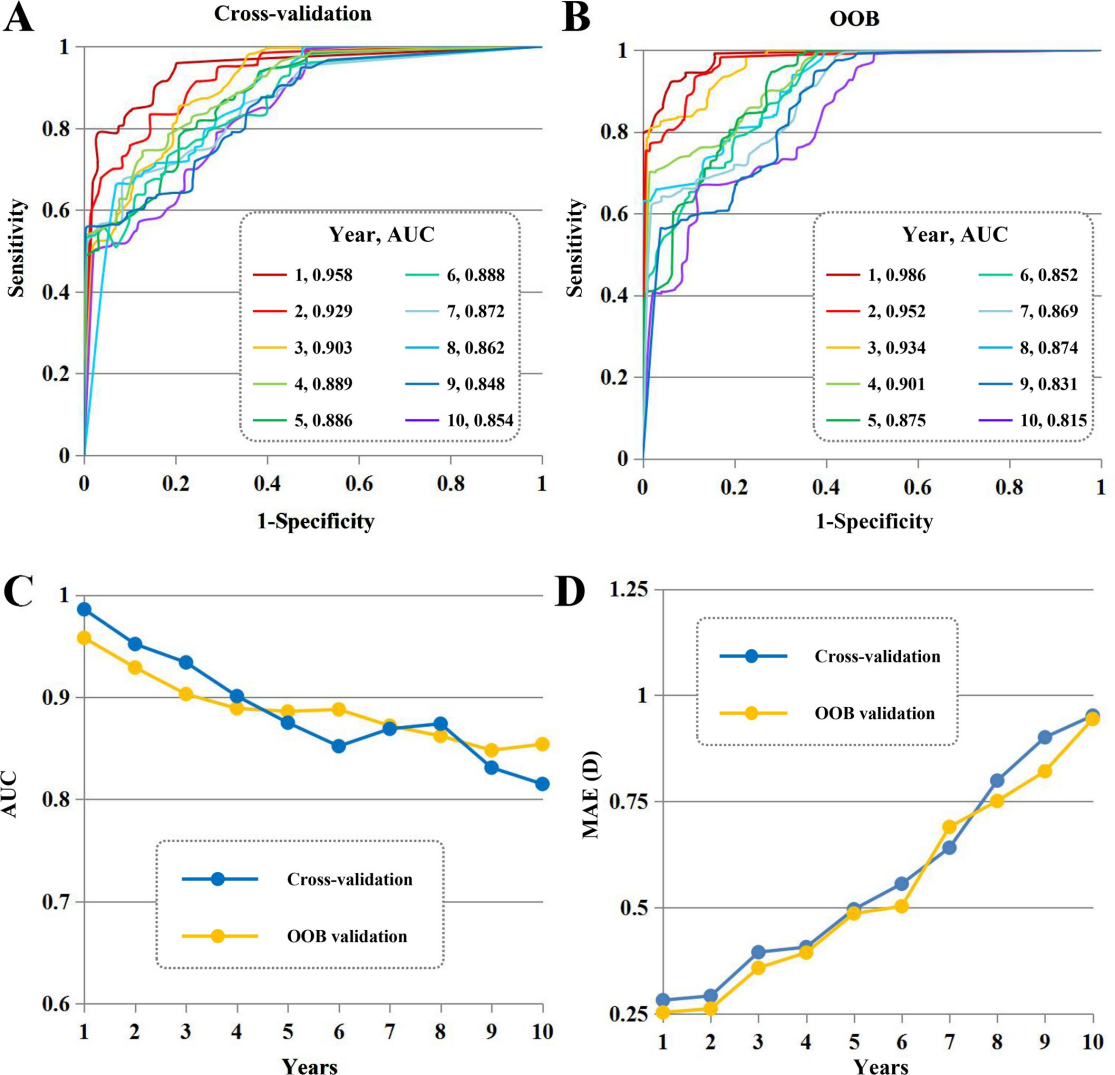
Algorithm performance in internal validation. (A and B) Our algorithm provided high-precision prediction for detecting high myopia in the cross-validation (the AUC ranged from 0.903 to 0.958 for 3 years, 0.886 to 0.889 for 5 years, and 0.862 to 0.888 for 8 years) and OOB tests (the AUC ranged from 0.934 to 0.986 for 3 years, 0.875 to 0.901 for 5 years, and 0.852 to 0.874 for 8 years). (C) AUC values more than 0.9 indicated excellent performance, and AUC values from 0.8 to 0.9 indicated good performance. Our algorithm provided excellent performance in the first 3 years and provided good performance in all 10 years. (D) MAE within 0.75 dioptres was considered a clinically acceptable prediction. Meanwhile, our algorithm achieved clinically acceptable prediction of the refraction value at time points after the initial baseline visit (the MAE ranged from 0.253 to 0.395 for 3 years, 0.394 to 0.496 for 5 years, and 0.503 to 0.799 for 8 years). AUC, area under the curve; D, dioptres; MAE, mean absolute error; OOB, out-of-bag.

As presented in Fig 3, our algorithm provided high-precision predictions of high myopia in the cross-validation (the AUC ranged from 0.903 to 0.958 for 3 years, 0.886 to 0.889 for 5 years, and 0.862 to 0.888 for 8 years) and OOB tests (the AUC ranged from 0.934 to 0.986 for 3 years, 0.875 to 0.901 for 5 years, and 0.852 to 0.874 for 8 years). Meanwhile, our algorithm achieved clinically acceptable prediction of the refraction value at each time point (year) after baseline assessment (the MAE ranged from 0.253 to 0.395 for 3 years, 0.394 to 0.496 for 5 years, and 0.503 to 0.799 for 8 years). The regressive performance and calibration curves of the algorithm are presented in Table 2 and S3 Fig, respectively. These calibration results also supported that our algorithm can predict the actual refraction values at time points over 10 years.

**Table 2:** Regressive performance of the algorithm using multi-resource datasets.

Analysis and dataset	Metric	Year									
		1	2	3	4	5	6	7	8	9	10
**Performance of the clinical prediction algorithm during internal validation**											
**Cross-validation**	***R***^**2**^	0.964	0.951	0.935	0.929	0.918	0.911	0.902	0.895	0.882	0.804
	**RMSE**	0.773	0.812	0.963	0.996	1.078	1.106	1.215	1.277	1.423	2.004
	**MAE**	0.282	0.292	0.395	0.407	0.496	0.556	0.641	0.799	0.901	0.952
**OOB**	***R***^**2**^	0.984	0.981	0.971	0.963	0.952	0.941	0.934	0.912	0.898	0.883
	**RMSE**	0.633	0.662	0.742	0.855	0.935	1.010	1.214	1.492	1.514	1.773
	**MAE**	0.253	0.262	0.358	0.394	0.486	0.503	0.690	0.751	0.821	0.944
**Performance of the multi-centre dataset during external validation**											
**DGC**	***R***^**2**^	0.952	0.948	0.924	0.884	0.851	0.834	0.811	0.802	0.759	0.728
	**RMSE**	0.797	0.866	0.881	0.967	1.273	1.414	1.517	1.553	1.708	1.878
	**MAE**	0.245	0.255	0.269	0.354	0.46	0.508	0.608	0.678	0.813	0.842
**HZD**	***R***^**2**^	0.949	0.928	0.903	0.885	0.844	0.819	0.795	0.743	0.708	0.699
	**RMSE**	0.812	0.824	0.892	1.056	1.322	1.455	1.781	1.904	2.019	2.406
	**MAE**	0.247	0.268	0.285	0.417	0.602	0.685	0.718	0.822	0.972	0.978
**PYD**	***R***^**2**^	0.972	0.963	0.949	0.889	0.851	—	—	—	—	—
	**RMSE**	0.623	0.795	0.814	1.131	1.845	—	—	—	—	—
	**MAE**	0.312	0.356	0.491	0.569	0.652	—	—	—	—	—
**HZC**	***R***^**2**^	0.968	0.942	0.939	0.903	0.833	0.812	—	—	—	—
	**RMSE**	0.812	0.901	0.962	1.094	1.364	1.578	—	—	—	—
	**MAE**	0.356	0.399	0.413	0.69	0.731	0.811	—	—	—	—
**HPD**	***R***^**2**^	0.994	0.981	0.952	0.926	0.922	0.910	0.890	0.860	—	—
	**RMSE**	0.594	0.615	0.801	0.994	1.114	1.264	1.419	1.815	—	—
	**MAE**	0.201	0.252	0.323	0.429	0.519	0.634	0.716	0.879	—	—
**HKC**	***R***^**2**^	0.929	0.904	0.897	—	—	—	—	—	—	—
	**RMSE**	0.787	0.846	1.077	—	—	—	—	—	—	—
	**MAE**	0.303	0.385	0.457	—	—	—	—	—	—	—
**LHD**	***R***^**2**^	0.916	0.888	—	—	—	—	—	—	—	—
	**RMSE**	0.818	1.233	—	—	—	—	—	—	—	—
	**MAE**	0.401	0.494	—	—	—	—	—	—	—	—
**Performance of the population-based dataset of the myopic study cohorts**											
**GOAL**	***R***^**2**^	0.899	0.852	0.744	—	—	—	—	—	—	—
	**RMSE**	0.507	0.702	1.136	—	—	—	—	—	—	—
	**MAE**	0.314	0.427	0.551	—	—	—	—	—	—	—
**RELS**	***R***^**2**^	0.884	0.832	0.774	0.722	—	—	—	—	—	—
	**RMSE**	0.516	0.633	0.847	1.505	—	—	—	—	—	—
	**MAE**	0.351	0.438	0.509	0.562	—	—	—	—	—	—

*R*^2^ values more than 0.950 are shaded dark green, *R*^2^ values from 0.900 to 0.950 are shaded medium green, *R*^2^ values from 0.800 to 0.899 are shaded light green, and *R*^2^ values less than 0.800 are shaded yellow.

DGC, Dongguan Guangming Ophthalmic Hospital; GOAL, Guangzhou Outdoor Activity Longitudinal Trial; HKC, Xiuying Optometry Department in Haikou City; HPD, Huangpu Optometry Department; HZC, Optometry Centre in Huizhou City; HZD, Haizhu Optometry Department; LHD, Haikou Longhua Optometry Department; MAE, mean absolute error; OOB, out-of-bag; PYD, Panyu Optometry Department; *R*^2^, coefficient of determination; RELS, Refractive Error Longitudinal Study; RMSE, root mean square error.

### External validation

**Fig 4:**
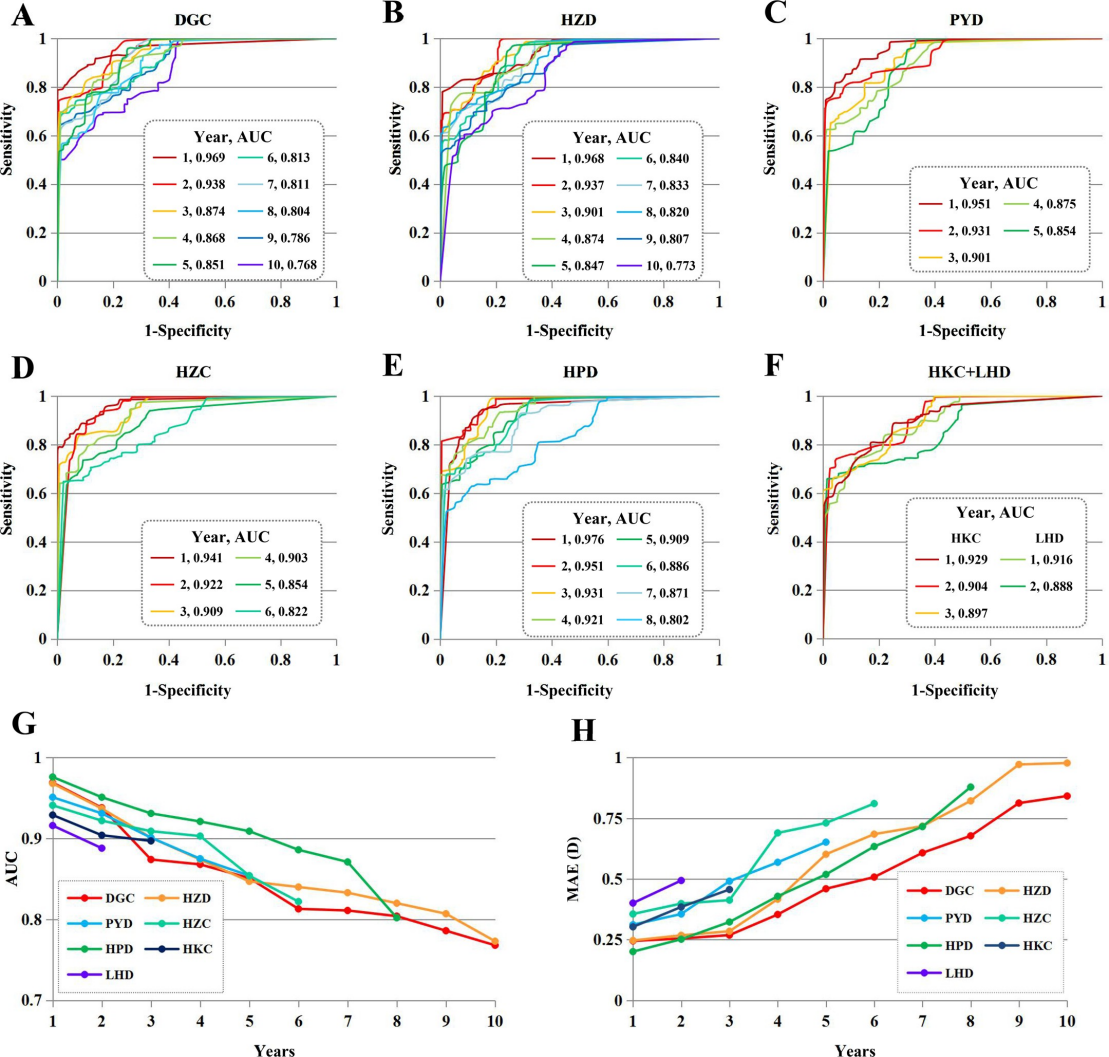
Algorithm performance in external validation. (A–F) Our algorithm achieved stable performance for high myopia detection in DGC (the AUC ranged from 0.768 to 0.969 for 10 years), the HZD (the AUC ranged from 0.773 to 0.968 for 10 years), the PYD (the AUC ranged from 0.854 to 0.951 for 5 years), the HZC (the AUC ranged from 0.822 to 0.941 for 6 years), the HPD (the AUC ranged from 0.802 to 0.976 for 8 years), the HKC (the AUC ranged from 0.897 to 0.929 for 3 years), and the LHD (the AUC ranged from 0.888 to 0.916 for 2 years). (G) Our algorithm provided excellent performance at 19 time points (43.18%, 19/44) and good performance at 22 time points (50.0%, 22/44). (H) A clinically acceptable prediction of the refraction value was provided at the majority of the time points examined (the MAE ranged from 0.201 to 0.494 for 3 years, 0.354 to 0.731 for 5 years, and 0.508 to 0.879 for 8 years). AUC, area under the curve; D, dioptres; DGC, Dongguan Guangming Ophthalmic Hospital; HKC, Xiuying Optometry Department in Haikou City; HPD, Huangpu Optometry Department; HZC, Optometry Centre in Huizhou City; HZD, Haizhu Optometry Department; LHD, Haikou Longhua Optometry Department; MAE, mean absolute error; PYD, Panyu Optometry Department.

The performance of the algorithm in the external validation is presented in Fig 4. Our algorithm achieved stable performance for high myopia detection in the DGC (the AUC ranged from 0.768 to 0.969 for 10 years), the HZD (the AUC ranged from 0.773 to 0.968 for 10 years), the PYD (the AUC ranged from 0.854 to 0.951 for 5 years), the HZC (the AUC ranged from 0.822 to 0.941 for 6 years), the HPD (the AUC ranged from 0.802 to 0.976 for 8 years), the HKC (the AUC ranged from 0.897 to 0.929 for 3 years), and the LHD (the AUC ranged from 0.888 to 0.916 for 2 years). Clinically acceptable prediction of the refraction value was achieved at the majority of the time points examined (the MAE ranged from 0.201 to 0.494 for 3 years, 0.354 to 0.731 for 5 years, and 0.508 to 0.879 for 8 years).

With respect to predicting the presence of high myopia (Table 3), our algorithm provided clinically acceptable prediction over 3 years (the AUC ranged from 0.940 to 0.985), 5 years (the AUC ranged from 0.856 to 0.901), and even 8 years (the AUC ranged from 0.801 to 0.837).

**Table 3:** Algorithm performance in predicting the presence of high myopia at 18 years of age.

Measure	Year									
	1	2	3	4	5	6	7	8	9	10
**AUC**	0.985	0.976	0.940	0.901	0.856	0.837	0.822	0.801	0.761	0.741
***n***	2,262	2,961	3,356	3,308	3,474	3,332	2,827	2,381	1,923	1,438
**Nn**	1,439	1,944	2,417	2,514	2,787	2,756	2,372	2,049	1,672	1,278
**Pn**	823	1,017	939	794	687	576	455	332	251	160

In the setting of predicting the presence of high myopia at 18 years old, our algorithm provided clinically acceptable prediction 3 years (the AUC ranged from 0.940 to 0.985), 5 years (the AUC ranged from 0.856 to 0.902), and even 8 years into the future (the AUC ranged from 0.801 to 0.837). AUC values more than 0.950 are shaded dark green, AUC values from 0.900 to 0.950 are shaded medium green, AUC values from 0.800 to 0.899 are shaded light green, and AUC values less than 0.800 are shaded yellow.

AUC, area under the curve; Nn, negative sample; Pn, positive sample (high myopia).

### Multi-resource test

**Fig 5:**
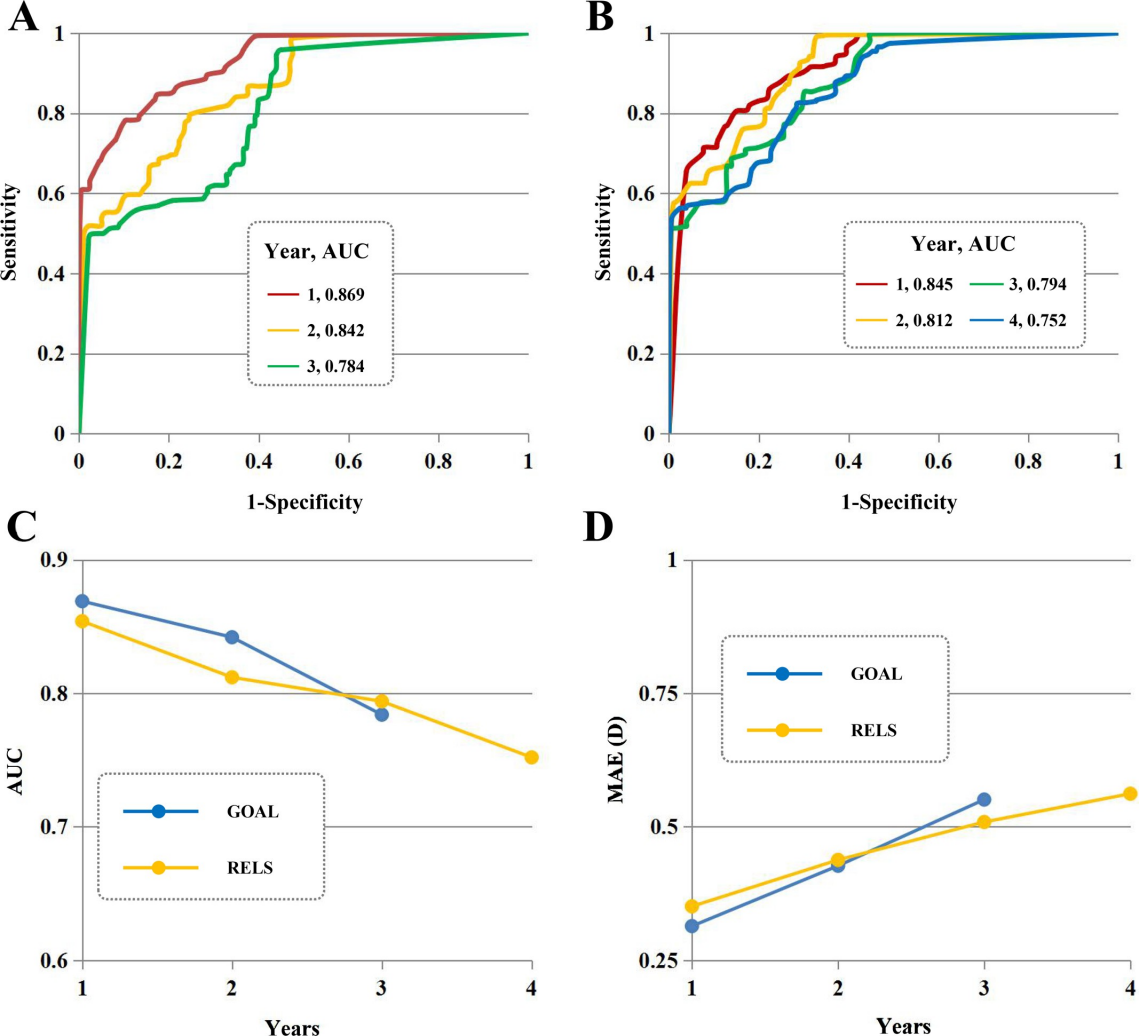
Algorithm performance in the multi-resource test. (A and B) Our algorithm presented stable high myopia detection in GOAL (the AUC ranged from 0.784 to 0.869 for 3 years) and RELS (the AUC ranged from 0.752 to 0.845 for 4 years). (C) Our algorithm provided excellent performance at 4 time points (57.1%, 4/7) and good performance at 3 time points (42.9%, 3/7). (D) A clinically acceptable prediction of refraction value was achieved at all time points examined (the MAE ranged from 0.314 to 0.562 for 4 years). AUC, area under the curve; D, dioptres; GOAL, Guangzhou Outdoor Activity Longitudinal Trial; MAE, mean absolute error; RELS, Refractive Error Longitudinal Study.

In the multi-resource test (Fig 5), our algorithm presented stable high myopia detection in GOAL (the AUC ranged from 0.784 to 0.869 for 3 years) and RELS (the AUC ranged from 0.752 to 0.845 for 4 years). A clinically acceptable prediction of refraction value was achieved at all time points examined (the MAE ranged from 0.314 to 0.562 for 4 years).

## Discussion

This study, to our knowledge for the first time, demonstrates the utilisation of large-scale electronic medical record data to generate a random forest algorithm for predicting disease prognosis, which, in our analysis, was the risk of developing high myopia in adulthood. Furthermore, this algorithm exhibited high accuracy in a predicting future trait, i.e., the dioptre value at 18 years of age. Our data suggest that this prediction can be performed as early as 8 years prior to an individual turning 18 years old.

Identifying “severe myopia” in younger children is of major clinical importance but poses a significant challenge. The severity of myopia is often estimated as the degree of SE, with an SE of −6.00 dioptres chosen as the cutoff to define high myopia. High myopia carries a much greater risk of developing other ocular complications, including retinal detachment, glaucoma, and pathological myopia [15,16]. Given that myopia is in the development phase during childhood, it is difficult to choose a specific SE cutoff to define “severe myopia” among children.

A few studies have identified children at a greater risk of progressive myopia [17–19]; however, none to our knowledge has attempted to predict actual SE or risk of high myopia in adulthood. The “risk classification” in previous studies was often inferred from the analysis of short-term longitudinal data, or a control group in the instance of intervention randomised trials. As such, the available data are only generalisable among children who meet the inclusion and exclusion criteria of the specific studies [20]. Due to pragmatic feasibility, most myopia control trials can only run for up to 3 years, and, similarly, longitudinal studies on myopia are often shorter than 5 years. Real-world electronic medical record data from established optometry services in tertiary ophthalmic centres are of considerable advantage in terms of the size of the dataset and the length of follow-up.

A prediction is meaningful only when it is accurate and early enough to provide an added clinical benefit. As demonstrated by our results, the accuracy of prediction is reduced when the targeted prediction time increases. However, interestingly, in our analysis, the accuracy, indicated by the AUC, remained high (0.80–0.90) for up to 8 years in both the internal and external validation. Furthermore, the 95% predicted dioptre of refraction was within 0.5 to 0.8 dioptres of the true value at 8 years. Such accurate “long-term” predictions are critically important given that current treatments for myopia control, including low-dose atropine [21] and orthokeratology lenses [22], are effective but often have potential side effects and therefore must be utilised effectively. In addition, accurate early prediction and timely treatment of myopia in its mild stages are important to maximise the treatment benefits.

Methodologically, the random forest algorithm, which is based on random selection and a combination of predictors [9], achieved superior performance in the current analysis compared to conventional methodologies (i.e., a generalised estimating equation and a mixed-effects model). The added value of the random forest algorithm presented a gradual enlargement after 3 years, indicating that myopia development became increasingly non-linear in long-term range. This advantage can be further appreciated in analyses that require the inclusion of more complex potential predictors in the model.

There are some limitations that must be addressed with the development of a prediction model using large-scale real-world clinical data. First, clinical data collected in real-world settings are often subject to bias, with compromised quality. For instance, although a standard clinical protocol was followed, refraction measurement was performed by a number of different optometrists in the present analysis. Despite this, one may argue that this “noise” can be de-emphasised by a stronger “signal” when the sample size is large enough. This effect has been highlighted in previous myopia genetic studies; for example, the genes identified by the Consortium for Refractive Error and Myopia (CREAM) study [23] (in which the refractive error was measured in every participant) were similar to those reported in the 23andMe study [24] (self-reported refractive error). Second, an algorithm developed from a training set may be subject to over-fitting, whereby the correlation or prediction is spurious [25]. This appears not to be the case in the present study, with our algorithm showing balanced contributions of all involved predictors and robust performance when evaluated in independent, external datasets.

In summary, to our knowledge, this study, for the first time, used large-scale data collected from the electronic health records from the largest ophthalmic centres in China to demonstrate the contribution of big data to the better prediction of disease prognosis. In the context of school-age myopia, the most prevalent eye disease in the Chinese population, our study demonstrates that machine learning prediction algorithms further translate the benefit of big data research into clinical practice. The performance of our predictive algorithm is promising, with large sample sizes and diversified data resources. This work proposes a novel direction for the use of medical big data mining to transform clinical practice and guide health policy-making and precise individualised interventions.

## Supporting information

S1 Code The source code of the algorithm training and validation. (PDF)

S1 Data The datasets of the Guangzhou Outdoor Activity Longitudinal Trial (GOAL) and the Refractive Error Longitudinal Study (RELS). (XLSX)

S1 Fig The variable contributions of the trained model on the entire Zhongshan Ophthalmic Centre (ZOC) dataset. The feature importance of spherical equivalent (SE), age at examination (AAE), and annual progression rate (APR) is presented. IncMSE, increase in mean square error.
(TIF)

S2 Fig Comparative performance of the random forest and other conventional algorithms. Based on the comparative analysis, the random forest (RF) algorithm outperforms the generalised estimating equation (GEE) and the mixed-effects model (ME) regarding the detection of high myopia.
(TIF)

S3 Fig Calibration curves in cross-validation. The mean differences of predicted and actual values at each time point are presented, with the interval as 0.25 dioptres (D) (the minimum interval of actual spherical equivalent [SE]). Our algorithm achieved stable prediction of refraction values over 10 years after baseline assessment in cross-validation.
(TIF)

S1 Table Demographic characteristics of the 2 population-based datasets. (DOCX)

S1 Text Detailed information on cohorts. (DOCX)

S2 Text Detailed information on out-of-bag validation. (DOCX)

S3 Text Detailed information on conventional algorithms. (DOCX)

## Abbreviations

AUC: area under the curve
DGC: Dongguan Guangming Ophthalmic Hospital
GOAL: Guangzhou Outdoor Activity Longitudinal Trial
HKC: Xiuying Optometry Department in Haikou City
HPD: Huangpu Optometry Department
HZC: Optometry Centre in Huizhou City
HZD: Haizhu Optometry Department
LHD: Haikou Longhua Optometry Department
MAE: mean absolute error
MSE: mean square error
OOB: out-of-bag
PYD: Panyu Optometry Department
RELS: Refractive Error Longitudinal Study
RMSE: root mean square error
SE: spherical equivalent
VIMP: variable importance
ZOC: Zhongshan Ophthalmic Centre
