# Supplementary material for: Prediction of myopia development among Chinese school-aged children using refraction data from electronic medical records: A retrospective, multicentre machine learning study
Source: PLoS Med. 2018 Nov 6;15(11):e1002674. doi: 10.1371/journal.pmed.1002674 (PMC6219762; doi:10.1371/journal.pmed.1002674)
Supplement: S1 Code — (PDF) [file pmed.1002674.s001.pdf]

S1 Code. The source code of the algorithms training and validation.

```
library(randomForest)
library(pROC)

#input ZOC data
ZOC<- read.csv("F: /ZOC.csv",header=TRUE)

#input Other 7 Centers data, GOAL, RELS
centers <- read.csv("F: /7centers.csv",header=TRUE)
GOAL <- read.csv("F: /GOAL.csv",header=TRUE)
RELS <- read.csv("F: /RELS.csv",header=TRUE)


#Training of ZOC data (Random forest algorithm)
#SE=targeted spherical equivalent; AAE=age at exam; APR=annual progression rate;
OSE=original spherical equivalent
set.seed(123)
rf_reg<-randomForest(SE~ AAE+APR+OSE,
                     data=ZOC,
                     mtry=2,ntree=500,
                     importance = T)

#10-fold cross validation
cv<- ZOC
set.seed(123)
folds<-createFolds(y= ZOC$SE,k=10)
max=0
num=0

fold<-data.frame()

for(i in 1:10){

  fold_test <- data[folds[[i]],]
  fold_train <- data[-folds[[i]],]

  fold_pre<-randomForest(SE~AAE+APR+OSE ,
                         data=fold_train,
                         mtry=2,ntree=500,
                         importance = T)

  fold_predict <- predict(fold_pre,newdata=fold_test)
```

```
    fold_test$predict = fold_predict

    fold_predict2 <- ifelse(fold_predict<=-6,1,0)

    fold_test$predict2 = fold_predict2

    fold<-rbind(fold,fold_test)
}
```

#Input 10-fold, OOB, Other 7 Centers data, GOAL, RELS in turn, to calculate index respectively

####1. Input 10-fold

```
rf.reg.pred<- fold$predict
data.test.new <- fold

pred.fore2<- fold$predict
rf.pred<-fold$predict2
```

####2. Input OOB

```
rf.reg.pred<- rf_reg$predicted
data.test.new <- ZOC

pred.fore2<- rf_reg$predicted
rf.pred<-ifelse(pred.fore2<=-6,1,0)
```

####3. Input Testing other 7 centers data, GOAL, RELS

#3.1 7centers

```
rf.reg.pred<- predict(rf_reg, newdata=centers)
data.test.new <- centers

pred.fore2<- predict(rf_reg, newdata=centers)
rf.pred<-ifelse(pred.fore2<=-6,1,0)
```

#3.2 GOAL

```
rf.reg.pred<- predict(rf_reg, newdata=GOAL)
data.test.new <- GOAL

pred.fore2<- predict(rf_reg, newdata=GOAL)
```

```
rf.pred<-ifelse(pred.fore2<=-6,1,0)
```

#3.3 RELS

```
rf.reg.pred<- predict(rf_reg, newdata=RELS)
data.test.new <- RELS

pred.fore2<- predict(rf_reg, newdata=RELS)
rf.pred<-ifelse(pred.fore2<=-6,1,0)
```

############
####Index###

#####Calculate R^2 RMSE MAE

```
degreenew <- list()
pred <- list()
Rsq <- vector()
Rmse <- vector()
Mae <- vector()
Mean <- vector()
L <- vector()


for (i in 1:10) {
  if (i==11){
    degreenew[[i]] <- data.test.new[data.test.new$time_dif>=i, ]$SE
    pred[[i]] <- rf.reg.pred[data.test.new$time_dif>=i]
  } else{
    degreenew[[i]] <- data.test.new[data.test.new$time_dif==i, ]$SE
    pred[[i]] <- rf.reg.pred[data.test.new$time_dif==i]
  }
  Rsq[i] <- 1-var(degreenew[[i]]-pred[[i]])/var(degreenew[[i]])
  Rmse[i] <- sqrt(mean((degreenew[[i]]-pred[[i]])^2))
  Mae[i] <- mean(abs(degreenew[[i]]-pred[[i]]))
  Mean[i] <- mean(abs(degreenew[[i]]))
  L[i] <- length(degreenew[[i]])
}

result <- as.data.frame(matrix(NA,length(L),6))
result[, 1:6] <- c(c(1:length(L)),Rsq,Rmse,Mae,Mean,L)
names(result) <- c('years','R2 ','RMSE','MAE','MEAN','n')
result <- t(result)
```

#####Calculate ROC AUC

```
auc<-vector()
t <- vector()
fp <- vector()
fn <- vector()
L <- vector()
p <- vector()
n <- vector()

for (i in 1:10) {
   if (i==11){
      pred <- rf.pred[data.test.new$time_dif >=i]
      y <- data.test.new[data.test.new$time_dif>=i, 'last']
      pred.p<-pred.fore2[data.test.new$time_dif>=i]
      roc.p<-roc(data.test.new[data.test.new$time_dif>=i, ]$last,pred.p,plot=FALSE)
   } else{
      pred <- rf.pred[data.test.new$time_dif==i]
      y <- data.test.new[data.test.new$time_dif==i, 'last']
      pred.p<-pred.fore2[data.test.new$time_dif==i]
      pred.p<-pred.fore2[data.test.new$time_dif==i]
      roc.p<-roc(data.test.new[data.test.new$time_dif==i, ]$last,pred.p,plot=FALSE)
   }
   t[i] <- prop.table(table(pred==y))[2]
   fp[i] <- sum(y==0&pred==1)/sum(y==0)
   fn[i] <- sum(y==1&pred==0)/sum(y==1)
   L[i] <- length(y)
   p[i] <- sum(y==1)
   n[i] <- sum(y==0)
   auc[i]<-roc.p$auc
}

result2 <- as.data.frame(matrix(NA,length(L),8))
result2[, 1:8] <- c(c(1:length(L)),t,fp,fn,L,p,n,auc)
names(result2) <- c("years","ACC","FPR","FNR","n","Pn","Nn","auc")
result2 <- t(result2)
```

####output index dataset in turn

```
write.csv(result,file="F: /result.csv")
write.csv(result2,file="F: /result2.csv")
```
