# Supplementary figures and images for: Prediction of myopia development among Chinese school-aged children using refraction data from electronic medical records: A retrospective, multicentre machine learning study
Source: PLoS Med. 2018 Nov 6;15(11):e1002674. doi: 10.1371/journal.pmed.1002674 (PMC6219762; doi:10.1371/journal.pmed.1002674)

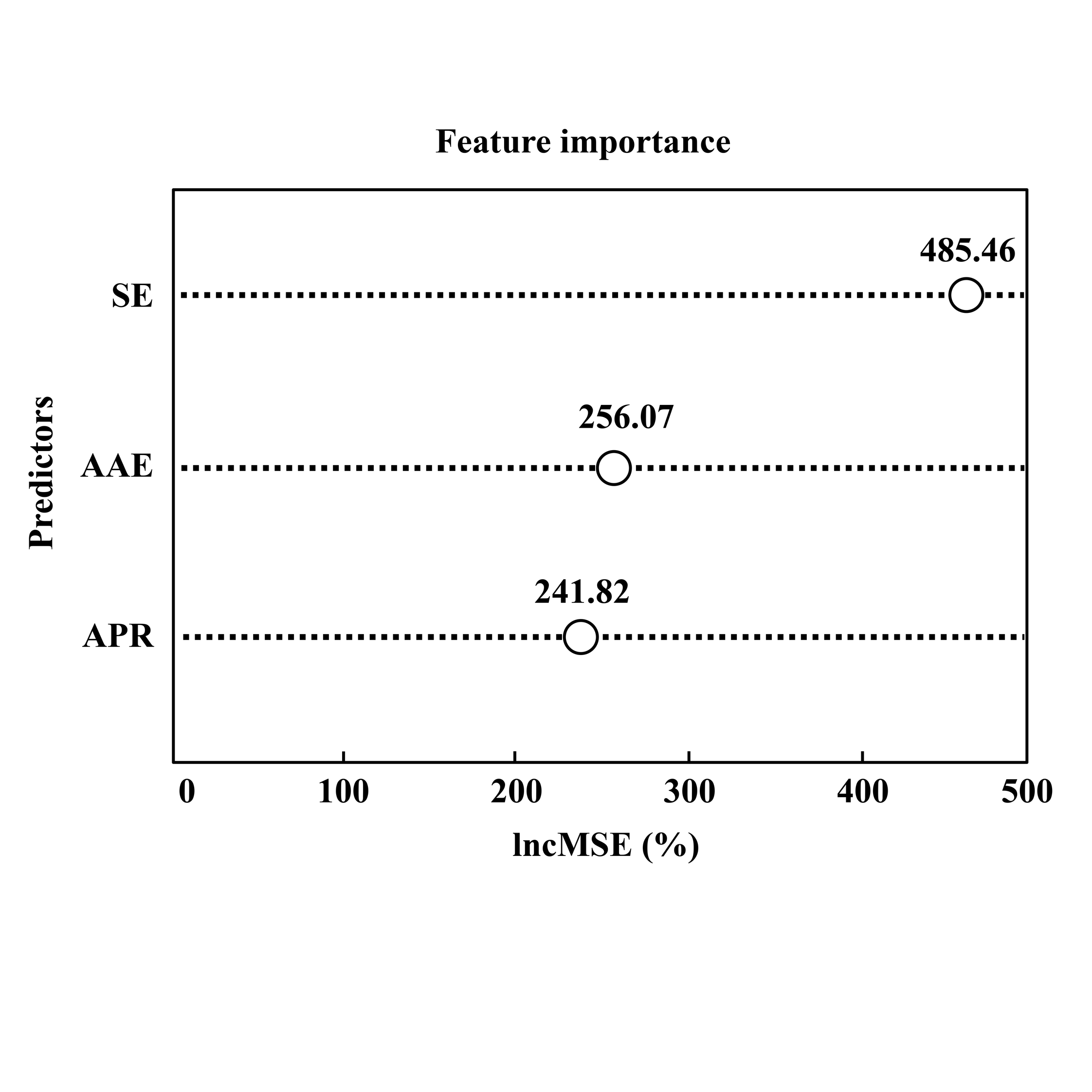

Supplement: S1 Fig — The feature importance of spherical equivalent (SE), age at examination (AAE), and annual progression rate (APR) is presented. IncMSE, increase in mean square error. (TIF) [file pmed.1002674.s003.tif]

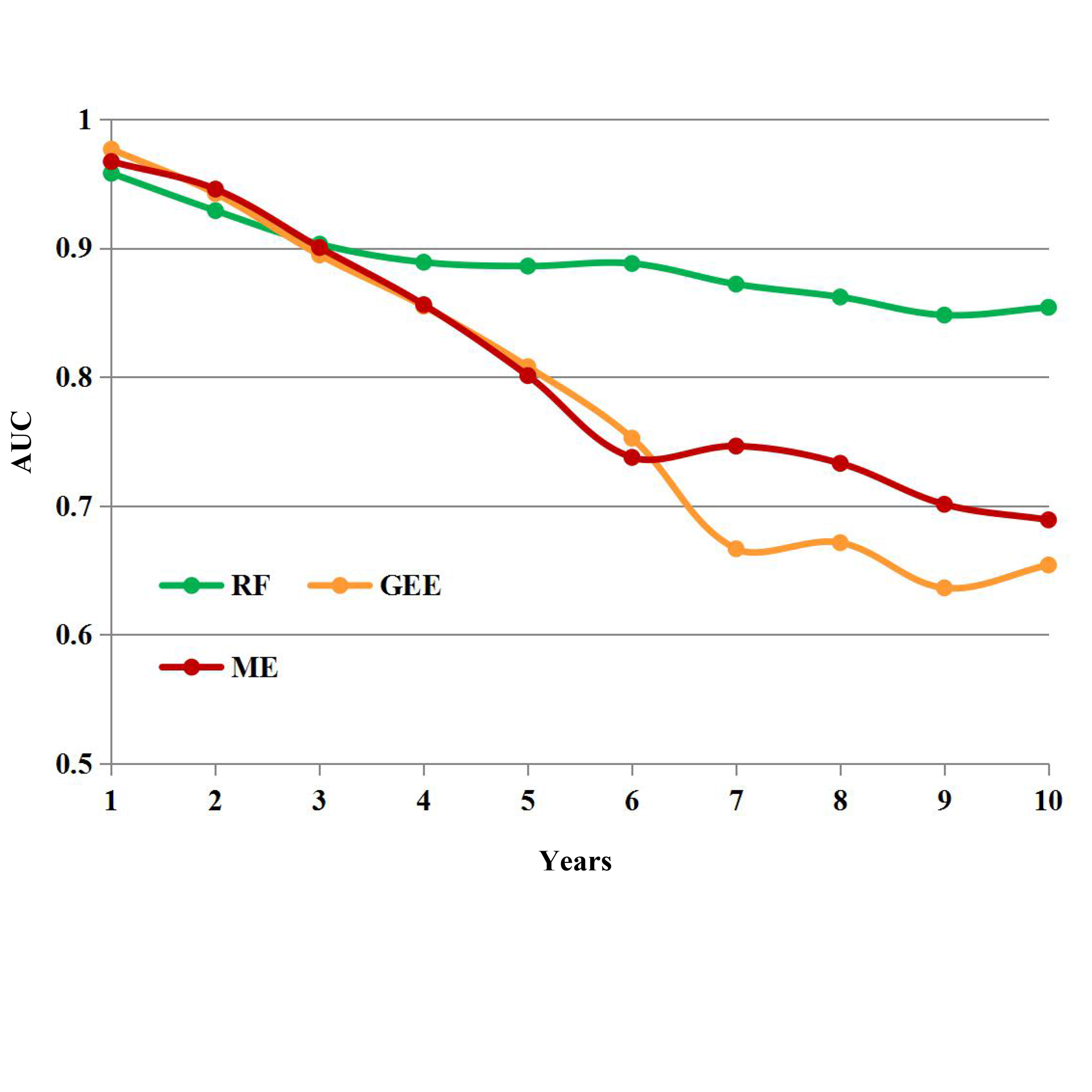

Supplement: S2 Fig — Based on the comparative analysis, the random forest (RF) algorithm outperforms the generalised estimating equation (GEE) and the mixed-effects model (ME) regarding the detection of high myopia. (TIF) [file pmed.1002674.s004.tif]

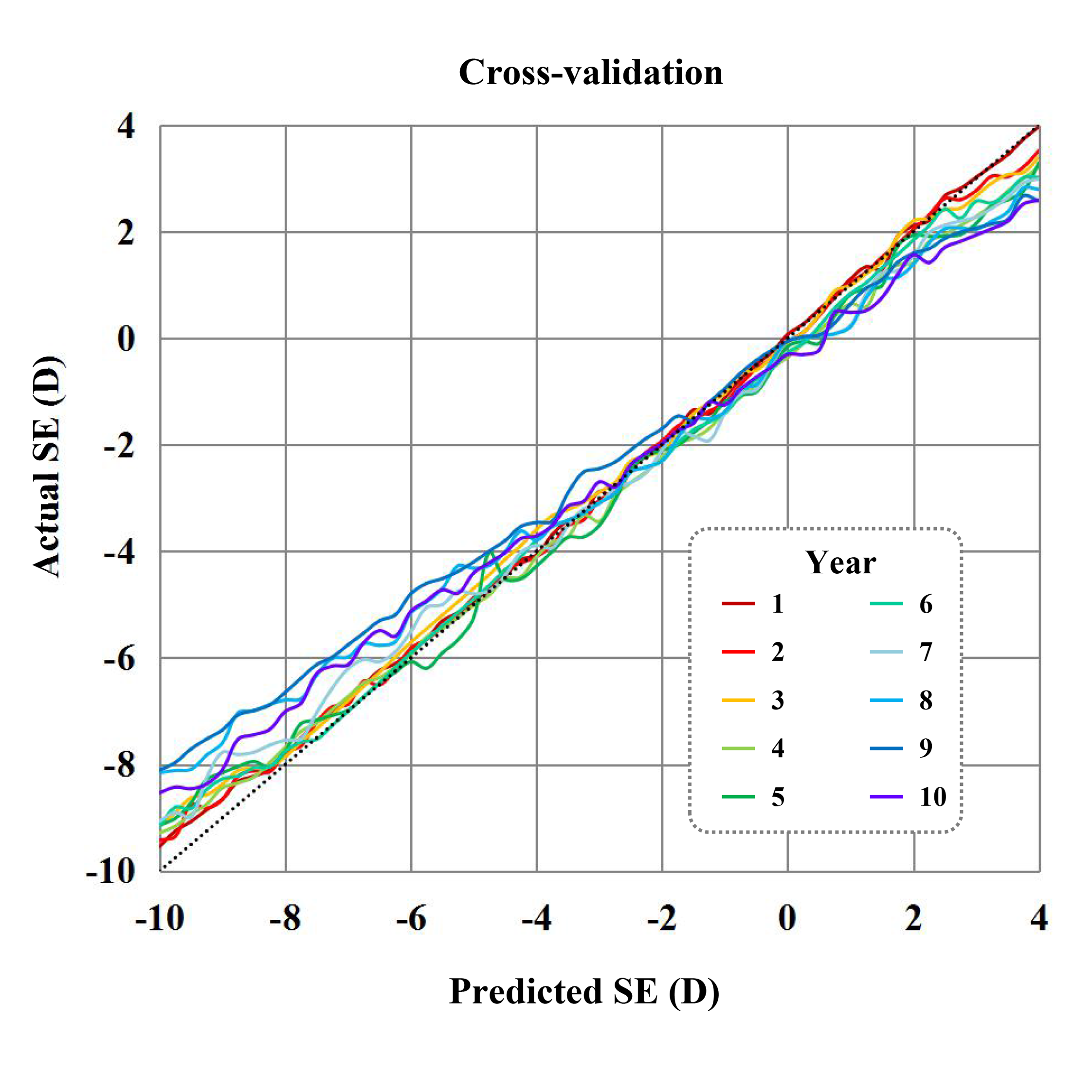

Supplement: S3 Fig — The mean differences of predicted and actual values at each time point are presented, with the interval as 0.25 dioptres (D) (the minimum interval of actual spherical equivalent [SE]). Our algorithm achieved stable prediction of refraction values over 10 years after baseline assessment in cross-validation. (TIF) [file pmed.1002674.s005.tif]
