# Supplementary material for: Prediction of myopia development among Chinese school-aged children using refraction data from electronic medical records: A retrospective, multicentre machine learning study
Source: PLoS Med. 2018 Nov 6;15(11):e1002674. doi: 10.1371/journal.pmed.1002674 (PMC6219762; doi:10.1371/journal.pmed.1002674)
Supplement: S1 Table — (DOCX) [file pmed.1002674.s006.docx]

**S1 Table.** **Demographic characteristics of the two population-based datasets.**

| **Guangzhou Outdoor Activity Longitudinal Trial (Mean±SD)** | | | | | |
| --- | --- | --- | --- | --- | --- |
| **Year of examination** | | **Number** | | **Age** | **Refraction**  **(dioptres)** |
|  |  | **(n)** | | **(years)** |  |
| **Baseline** | | 1609 | | 6.59±0.32 | 1.28±0.90 |
| **1^st^ year** | | 1547 | | 7.57±0.32 | 0.87±1.10 |
| **2^nd^ year** | | 1549 | | 8.57±0.32 | 0.32±1.33 |
| **3^rd^ year** | | 1525 | | 9.67±0.40 | -0.23±1.62 |
| **Refractive Error Longitudinal Study of Chinese children (Mean±SD)** | | | | | |
| **Baseline** | 2030 | | 7.19±0.37 | | 0.30±0.99 |
| **1^st^ year** | 1870 | | 8.16±0.36 | | -0.17±0.97 |
| **2^nd^ year** | 1829 | | 9.16±0.36 | | -0.51±1.20 |
| **3^rd^ year** | 1803 | | 10.12±0.36 | | -0.87±1.41 |
| **4^th^ year** | 1661 | | 11.08±0.35 | | -1.28±1.64 |
| **5^th^ year** | 1690 | | 12.15±0.35 | | -1.71±1.87 |

**Footnotes:** SD=standard deviation.
