# Supplementary material for: Prediction of myopia development among Chinese school-aged children using refraction data from electronic medical records: A retrospective, multicentre machine learning study
Source: PLoS Med. 2018 Nov 6;15(11):e1002674. doi: 10.1371/journal.pmed.1002674 (PMC6219762; doi:10.1371/journal.pmed.1002674)
Supplement: S1 Text — (DOCX) [file pmed.1002674.s007.docx]

**S1 Text. Detailed information of cohorts**

The first cohort data were obtained from the GOAL Trial between October 2010 and October 2013. School-aged children in grades 1 to 12 of primary schools in Guangzhou were clustered randomly into two groups. Children with tropia, amblyopia, and intellectual disabilities were excluded from the study. All eligible participants received cycloplegic refraction annually after obtaining consent from their legal guardian.

Another cohort dataset was drawn from the RELS in Chinese children. This study enrolled children in grade 1 (6-7 years old) from 19 primary schools classified as “surveillance” schools by the Guangzhou Ministry of Education. Follow-up examinations were conducted annually after enrolment until the participants graduated from primary school. The purpose of this study was to describe the presence and progression of school myopia in children and to better understand the risk factors pertaining to myopia development based on an annual prospective follow-up examination of a homogeneous age (approximately 6 years). Students with any ocular diseases that would cause visual acuity impairment as well as those who could not provide written consent from their parents or guardians were excluded. The cycloplegic refraction was included in the annual examinations.
