# Supplementary material for: Prediction of myopia development among Chinese school-aged children using refraction data from electronic medical records: A retrospective, multicentre machine learning study
Source: PLoS Med. 2018 Nov 6;15(11):e1002674. doi: 10.1371/journal.pmed.1002674 (PMC6219762; doi:10.1371/journal.pmed.1002674)
Supplement: S2 Text — (DOCX) [file pmed.1002674.s008.docx]

**S2 Text. Detailed information out-of-bag (OOB) validation.**

The OOB validation was performed internally using the error estimate as follows: in each bootstrap sampling, some of the training samples were omitted and thus not used in the construction of the decision tree. Subsequently, as the forest was built, each tree was tested on samples not used in building that tree, which were called OOB samples. Each OOB sample was moved down the tree to obtain a predicted value and a classified label. At the end of the run, the mean predicted value and the majority of the classified label over all of the involved trees were the algorithm-predicted results for the OOB sample.
