# Supplementary material for: Prediction of myopia development among Chinese school-aged children using refraction data from electronic medical records: A retrospective, multicentre machine learning study
Source: PLoS Med. 2018 Nov 6;15(11):e1002674. doi: 10.1371/journal.pmed.1002674 (PMC6219762; doi:10.1371/journal.pmed.1002674)
Supplement: S3 Text — (DOCX) [file pmed.1002674.s009.docx]

**S3 Text. Detailed information of conventional algorithms.**

**Generalized estimating equation**

In statistics, the generalized estimating equation (GEE) is an extension of generalized linear models that provides a semi-parametric approach to the longitudinal analysis of categorical and continuous responses. The GEE approach can be used to estimate the parameters of a generalized linear model with an unknown correlation for the within-subject association among the repeated measurements. This classic method is commonly used in longitudinal studies due to its ability to handle various types of unmeasured dependence among the repeated measurements and its consistent estimation, even with a mis-specified correlation structure.

**Mixed-effects model**

The mixed-effects model is a conventional alternative for longitudinal data that contains both fixed effects and random effects. The fixed effects are analogous to regression coefficients and are estimated directly by assuming common relationships between the outcomes and covariates for all subjects. The random effects indicate how much the subject specific profiles deviate from the population mean due to individual differences; these differences are not directly estimated but are summarized according to their estimated fixed effects and covariance structure. This approach is widely used in analysing longitudinal data because it can handle uneven spacing of repeated measurements and provide an estimated trajectory for each individual.
